# Supplementary material for: Taste bud formation depends on taste nerves
Source: eLife. 2019 Oct 1;8:e49226. doi: 10.7554/eLife.49226 (PMC6785267; doi:10.7554/eLife.49226)
Supplement: Figure 2—source data 1. [file elife-49226-fig2-data1.docx]

**Source data for Figure 2**

Raw data of the measure of the cross-sectional area (in μm^2^) occupied per taste bud (occasionally 1 to 3 taste buds in the same miscrcoscopic field, as indicated) in the soft palate of 3 wild type mice and 3 *Neurog2* KO mice at E18.5.


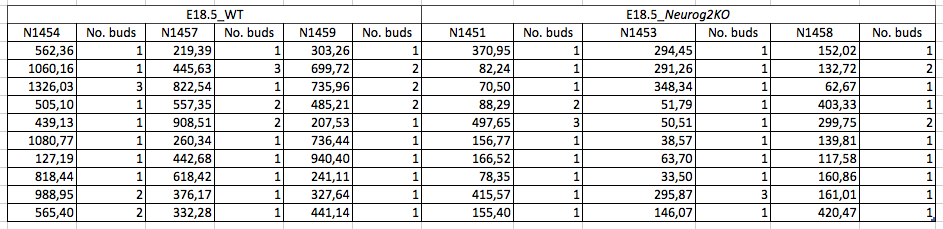


Raw data of the measure of the surface occupied per taste bud in the soft palate of 4 wild type mice and 4 *Neurog2* KO mice at E20.5.


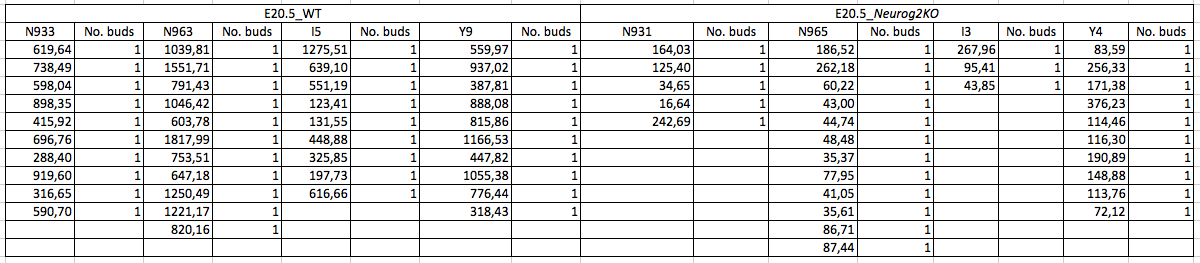


Raw data of the measure of the surface occupied per taste bud in the soft palate of 3 wild type mice and 3 *Neurog1* KO mice at E20.5.


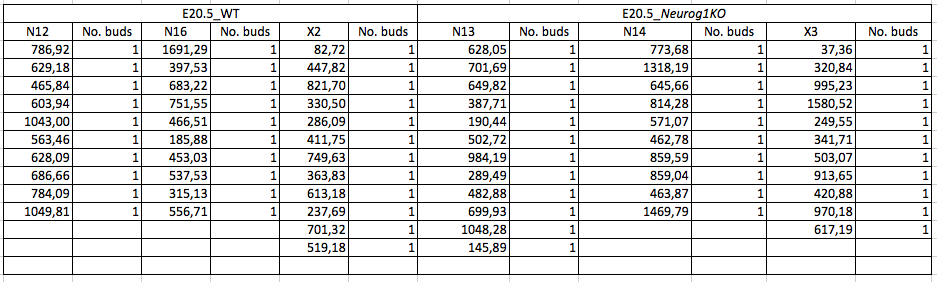


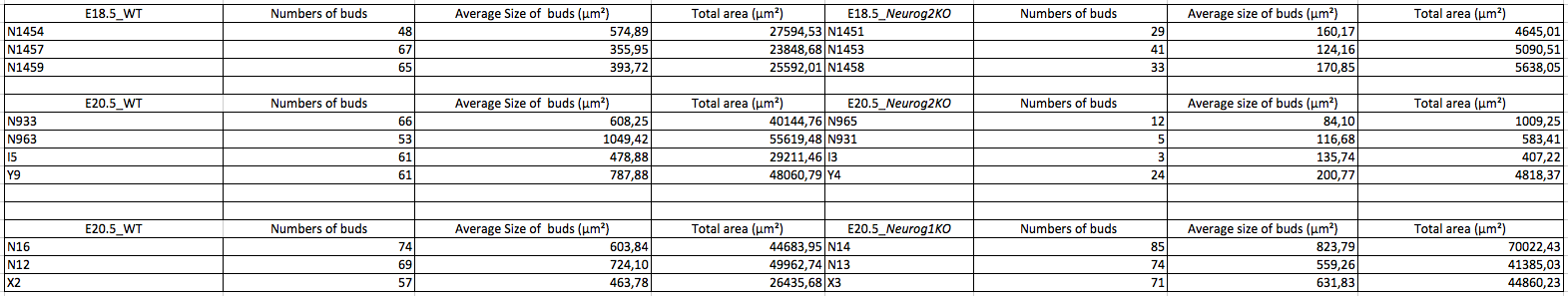


Raw data of the number of taste buds, average size and total size in the hemi-soft palate of E18.5 *Neurog2* KO, E20.5 *Neurog2* KO and *Neurog1* KO and their wild type littermates.
