# Supplementary material for: Taste bud formation depends on taste nerves
Source: eLife. 2019 Oct 1;8:e49226. doi: 10.7554/eLife.49226 (PMC6785267; doi:10.7554/eLife.49226)

**Source data for Figure 4**

Number of Sox2^+^ cells for each K8^+^ cell clusters at E18.5 in wild type (N=3) and *Neurog2*KO (N=3)


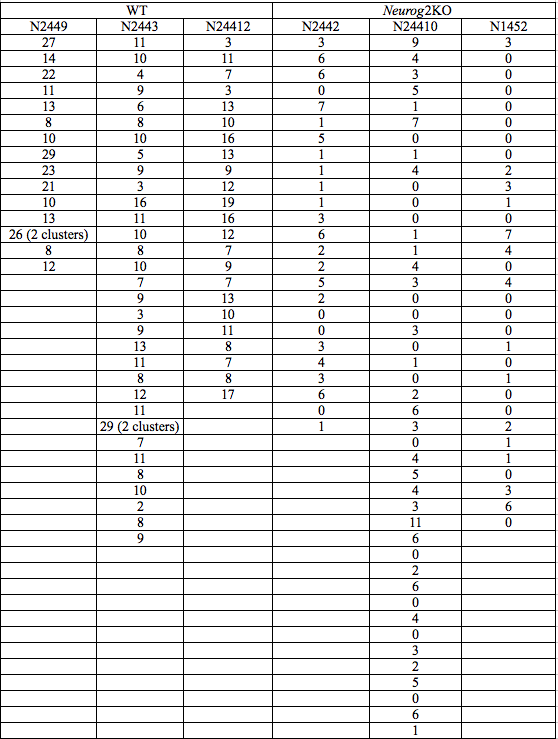


Number of Ki67^+^ cells for each K8^+^ cell clusters at E18.5 in wild type (N=3) and *Neurog2*KO (N=3)


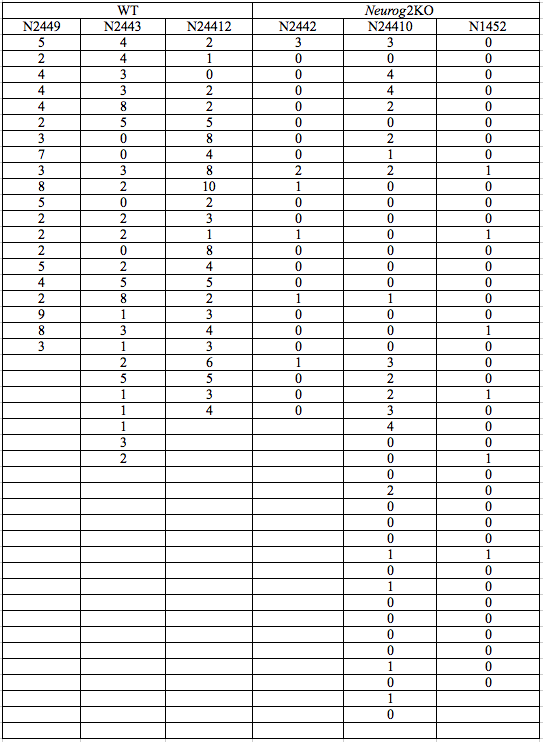

Supplement: Figure 4—source data 1. [file elife-49226-fig4-data1.docx]
