# Supplementary material for: Taste bud formation depends on taste nerves
Source: eLife. 2019 Oct 1;8:e49226. doi: 10.7554/eLife.49226 (PMC6785267; doi:10.7554/eLife.49226)

**Source data for Material and Methods**

Average width of K8^+^ cell clusters in wild type and *Neurog2*KO at E18.5 and E20.5.


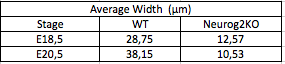


Width of each K8^+^ cell clusters at E18.5 (wild type, N=3; *Neurog2*KO, N=3) and E20.5 (wild type, N=4; *Neurog2*KO, N=4), respectively


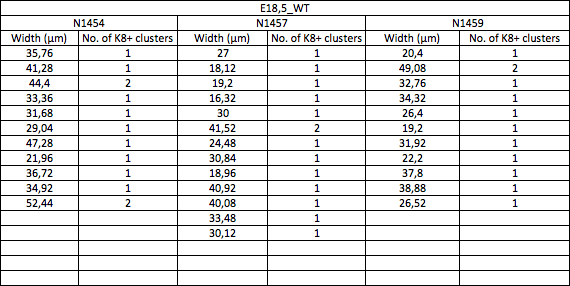


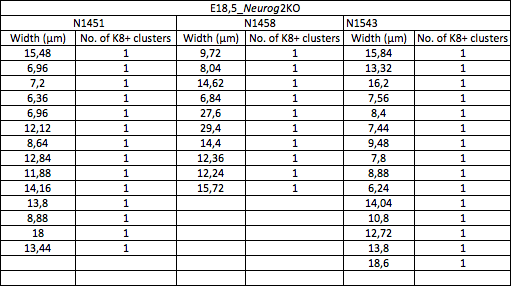


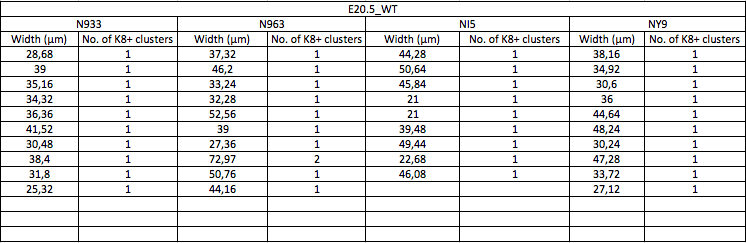


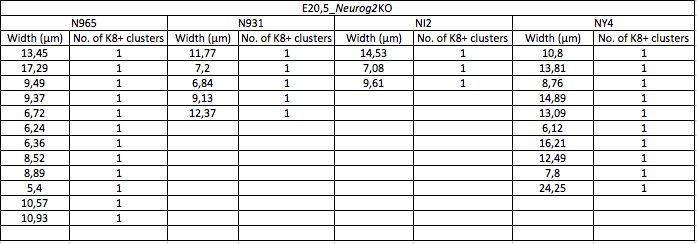

Supplement: Source data 1. [file elife-49226-data1.docx]
